# Hydrothermal synthesis of dittmarite-group NH_4_(Co_1−*x*_Mn_*x*_)PO_4_·H_2_O particles as inorganic violet pigments†

**DOI:** 10.1039/d3ra07387d

**Published:** 2024-01-08

**Authors:** Hiroaki Uchiyama, Saori Tachibana

**Affiliations:** a Department of Chemistry and Materials Engineering, Kansai University 3-3-35 Yamate-cho Suita 564-8680 Japan h_uchi@kansai-u.ac.jp +81-6-6368-1121 ext. 6131; b Kansai University Japan

## Abstract

Dittmarite-group NH_4_(Co_1−*x*_Mn_*x*_)PO_4_·H_2_O particles were prepared *via* a hydrothermal route. Single-phase platelike NH_4_(Co_1−*x*_Mn_*x*_)PO_4_·H_2_O particles were obtained from aqueous solutions containing MnCl_2_·4H_2_O, CoCl_2_·6H_2_O, and (NH_4_)_2_HPO_4_, where the [Mn^2+^]/([Co^2+^] + [Mn^2+^]) mole ratios in the products were controlled by changing the MnCl_2_ and CoCl_2_ concentrations of the precursor solutions. The vivid violet colour of the ammonium cobalt phosphate (NH_4_CoPO_4_·H_2_O) particles was maintained upon substitution of Co^2+^ with Mn^2+^ ions up to *x* = 0.8, thus achieving an 80% saving of cobalt in the preparation of violet pigments.

## Abstract 2

Dittmarite-group NH_4_(Co_1−*x*_Mn_*x*_)PO_4_·H_2_O particles were prepared *via* a hydrothermal route. The violet colour of the particles was maintained upon substitution of Co^2+^ with Mn^2+^ ions up to *x* = 0.8, thus achieving an 80% saving of cobalt.

## Graphical abstract

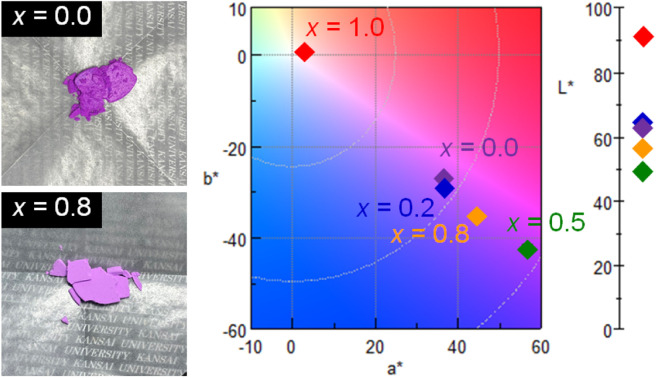

## Introduction

Dittmarite-group minerals, which have the general formula ABPO_4_·H_2_O (A = NH_4_^+^ or K^+^, B = Mn^2+^, Co^2+^, Mg^2+^, Ni^2+^), have received significant research attention for various applications, such as pigments, catalysts, fertilizers, and magnetic devices.^1–3^ The device properties of dittmarite-group materials are determined by the cation species, and thus synthetic routes that enable compositional control are strongly required.

Cobalt phosphate materials, which exhibit various colour hues from pinkish purple to deep violet depending on their composition and crystal phase characteristics, are widely used as inorganic pigments.^4–7^ Ammonium cobalt phosphate monohydrate (NH_4_CoPO_4_·H_2_O), which is a member of the dittmarite group, has been identified as the main component in the pigment cobalt violet light (C.I. 77362 PV49).^4^ In recent years, NH_4_CoPO_4_·H_2_O has also been investigated as an electrocatalyst for the oxygen evolution reaction,^8,9^ a precursor of LiCoPO_4_ cathode materials for Li-ion batteries,^10,11^ and a high-performance supercapacitor.^12–14^ Accordingly, ammonium cobalt phosphate materials have drawn extensive research attention. However, cobalt is expensive, toxic, and susceptible to supply-chain instability, necessitating the development of alternative materials.^15–17^

Manganese phosphate minerals are also well known as inorganic violet pigments.^18–21^ Manganese violet is a typical commercial manganese phosphate pigment, the principal components of which have been identified as α- and β-NH_4_MnP_2_O_7_.^18^ Niahite (NH_4_MnPO_4_·H_2_O) is a dittmarite-group manganese material that occurs naturally in guano deposits.^22^ Synthetic routes to niahite have been studied recently owing to its great application potential for magnetic devices,^23^ supercapacitors,^24,25^ and as a precursor of olivine-type cathodes for Li-ion batteries.^26,27^ However, its potential applications to pigments and jewellery have not been fully investigated.

Because of their similarity in ionic radius, Mn^2+^ ions can partially replace Co^2+^ ions in the dittmarite crystal lattice, resulting in the formation of binary compounds represented by the formula NH_4_(Co_1−*x*_Mn_*x*_)PO_4_·H_2_O. Manganese is less toxic and more available than cobalt.^28–30^ Therefore, the substitution of Co^2+^ by Mn^2+^ could provide a means of cobalt resource saving and cost reduction^30,31^ without loss of function. The compositional control of dittmarite-group NH_4_(Co_1−*x*_Mn_*x*_)PO_4_·H_2_O phase would be useful for researchers in not only pigment but also many other material fields such as catalysts,^8,9^ capacitors,^12–14,24,25^ magnetic devises^23^ and electrodes for Li-ion batteries.^10,11,26,27^

In this work, we prepared NH_4_(Co_1−*x*_Mn_*x*_)PO_4_·H_2_O particles *via* a hydrothermal route and evaluated their colour variation with the replacement of Co^2+^ ions by Mn^2+^ ions to different degrees. The binary dittmarite-group compounds were obtained from aqueous solutions containing MnCl_2_·4H_2_O, CoCl_2_·6H_2_O, and (NH_4_)_2_HPO_4_ by hydrothermal treatment. The effects of compositional changes on the colour characteristics of the products were quantitatively evaluated in terms of L*a*b* colour parameters, as calculated from UV-Vis absorption spectra.

## Experimental

CoCl_2_·6H_2_O (∼99.0 mass%, Wako Pure Chemical Industries, Osaka, Japan) (0–0.48 g) and MnCl_2_·4H_2_O (∼99.0 mass%, Wako Pure Chemical Industries, Osaka, Japan) (0–0.40 g) were dissolved in 20 mL purified water at room temperature under stirring ([CoCl_2_·6H_2_O] = 0–0.10 M, [MnCl_2_·4H_2_O] = 0–0.10 M). The total amount of Co^2+^ and Mn^2+^ ions in the solutions was fixed at 0.10 M ([CoCl_2_·6H_2_O] + [MnCl_2_·4H_2_O] = 0.10 M), and the [Mn^2+^]/([Co^2+^] + [Mn^2+^]) mole ratios were denoted as *x* (*x* = 0–1.0). (NH_4_)_2_HPO_4_ solutions were prepared by addition of (NH_4_)_2_HPO_4_ (0.53 g) to 20 mL of purified water ([(NH_4_)_2_HPO_4_] = 0.20 M). The metal–salt solution containing CoCl_2_ and MnCl_2_ was poured into the (NH_4_)_2_HPO_4_ solution. The mixed solution immediately became cloudy. After stirring for 1 h, the cloudy suspension was treated hydrothermally at 150 °C in a Teflon-lined stainless steel autoclave (75 mL, Flon Industry, Tokyo, Japan) for 1 day. NH_4_(Co_1−*x*_Mn_*x*_)PO_4_·H_2_O samples were obtained by washing the resulting precipitates with purified water and drying at 60 °C for 1 day.

The samples were observed with an optical microscope (KH-1300, HiROX, Tokyo, Japan). The crystalline phases of the products were identified by powder X-ray diffraction (XRD) in normal 2*θ*/*θ* mode using an X-ray diffractometer (MiniFlex 600, Rigaku, Tokyo, Japan) with Cu Kα radiation at 40 kV and 15 mA. The morphologies of the products were investigated by scanning electron microscopy (SEM; JSM-6510, JEOL, Tokyo, Japan). The chemical compositions of the samples were determined by X-ray photoelectron spectroscopy (XPS; PHI5000 Versa Probe, ULVAC-PHI, Chigasaki, Japan) with a monochromatic Al Kα X-ray source. A charge neutralizer was used to counter surface charging during collection of the spectra. Diffuse-reflectance UV-Vis absorption spectra of the samples were obtained using an optical spectrometer (V-770, JASCO, Tokyo, Japan) with an integrating sphere, and the colour parameters in L*a*b* colour space were calculated from the absorption spectra. a*, b*, and L* values denote the red intensity (green-to-red axis), yellow intensity (blue-to-yellow axis), and luminosity, respectively, of a sample.

The heat resistance of the samples were evaluated by thermogravimetric and differential thermal analysis (TG-DTA) curves those were obtained at a heating rate of 10 °C min^−1^ in flowing air using a thermal analyzer (ThermoPlus 2, Rigaku, Tokyo, Japan). The chemical resistance was evaluated based on Japanese Industrial Standards (JIS); K5101-8:2004, where 0.1 g of the samples were dipped in 20 mL of 2 wt% HCl and 2 wt% NaOH aqueous solutions for 30 min at room temperature and then the colour change of the samples was visually checked.

## Results and discussion

**Fig. 1:**
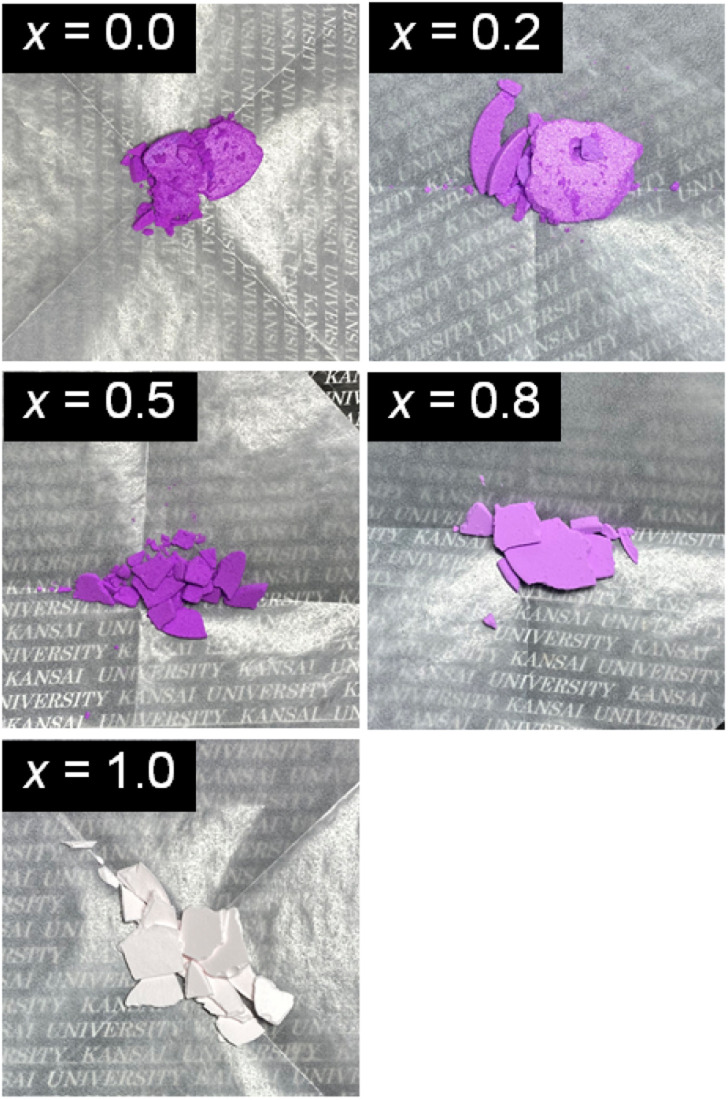
Appearances of NH_4_(Co_1−*x*_Mn_*x*_)PO_4_·H_2_O samples with *x* = 0–1.0.

Fig. 1 shows the appearances of the NH_4_(Co_1−*x*_Mn_*x*_)PO_4_·H_2_O products with *x* = 0–1.0. Violet-coloured precipitates were obtained for *x* = 0–0.8, while light-pink-coloured products were produced for *x* = 1.0. The oxidation states and the molar ratios of Co and Mn ions in the products were determined by XPS analysis. The XPS spectra are shown in ESI Fig. S1 and S2.† Only peaks attributed to bivalent Co^2+^ and Mn^2+^ ions are observed for the NH_4_(Co_1−*x*_Mn_*x*_)PO_4_·H_2_O samples. The [Mn^2+^]/([Co^2+^] + [Mn^2+^]) mole ratios in the samples (*x*′) are shown in Table 1. The Mn^2+^ contents in the samples (*x*′) increase linearly with those in the precursor solutions (*x*), with the *x*′ values being slightly lower than *x*.

**Table 1:** The [Mn^2+^]/([Co^2+^] + [Mn^2+^]) mole ratio of the precursor solutions (*x*) and the samples (*x*′) measured by XPS analysis

[Mn^2+^]/([Co^2+^] + [Mn^2+^]) mole ratio	
*x* (precursor solutions)	*x*′ (samples)
0.0	—
0.2	0.18
0.5	0.46
0.8	0.65
1.0	—

**Fig. 2:**
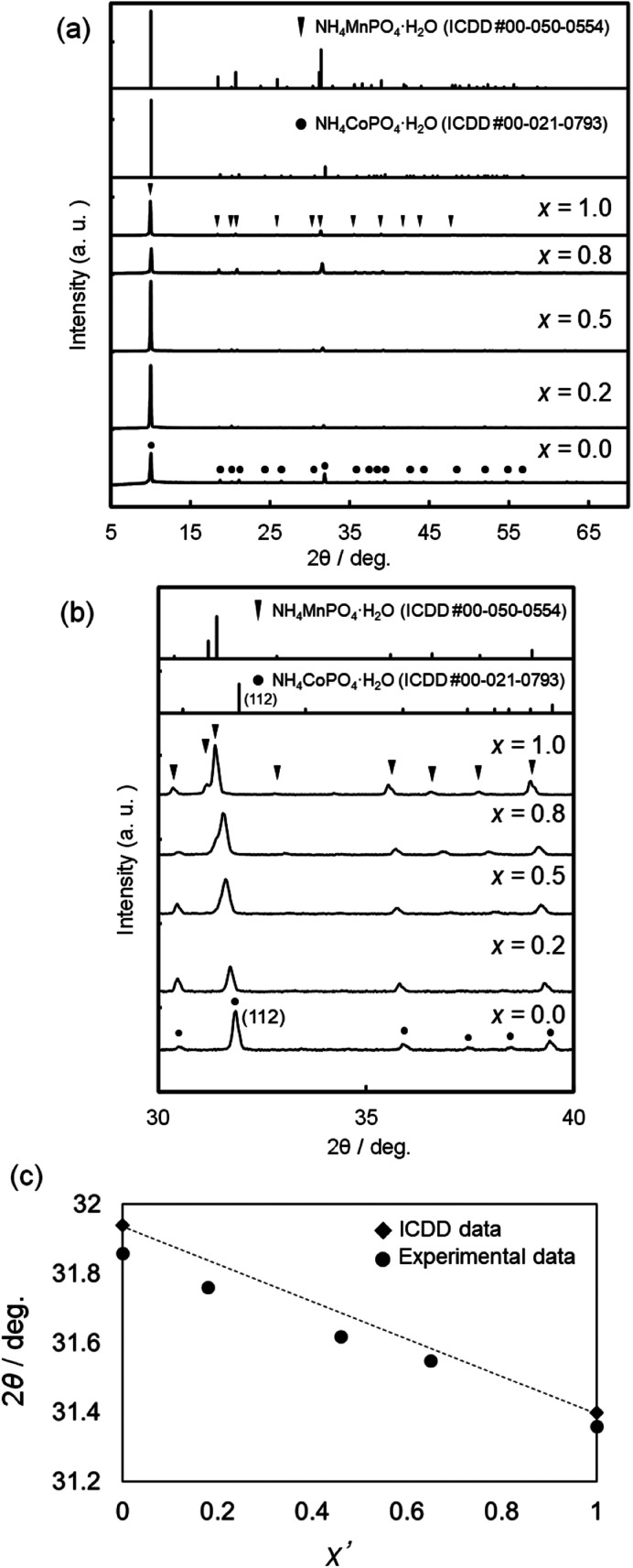
XRD patterns of NH_4_(Co_1−*x*_Mn_*x*_)PO_4_·H_2_O samples with *x* = 0–1.0. Wide- (a) and narrow-range (b) XRD patterns, and the relationship between Mn content (*x*′) and the diffraction angle of the (112) face (c).

Fig. 2 shows the XRD patterns of NH_4_(Co_1−*x*_Mn_*x*_)PO_4_·H_2_O samples with *x* = 0–1.0. Diffraction peaks attributed to ammonium cobalt phosphate (NH_4_CoPO_4_·H_2_O) are observed for *x* = 0, while single-phase ammonium manganese phosphate (NH_4_MnPO_4_·H_2_O) is observed for *x* = 1.0 (Fig. 2a). For the binary samples with *x* = 0.2–0.8, only the diffraction peaks attributed to single-phase dittmarite were also observed (Fig. 2a), where the peaks shift to lower angles with increasing *x* between NH_4_CoPO_4_·H_2_O (*x* = 0) and NH_4_MnPO_4_·H_2_O (*x* = 1.0) phases (Fig. 2b). The relationship between the Mn contents in the samples (*x*′) and the (112) diffraction angles are shown in Fig. 2c. The (112) diffraction angles decrease linearly with *x*′. These results could indicate the formation of NH_4_(Co_1−*x*_Mn_*x*_)PO_4_·H_2_O solid-solution phases and the [Mn^2+^]/([Co^2+^] + [Mn^2+^]) mole ratios in the products changed with the MnCl_2_ and CoCl_2_ concentrations of the precursor solutions.

**Fig. 3:**
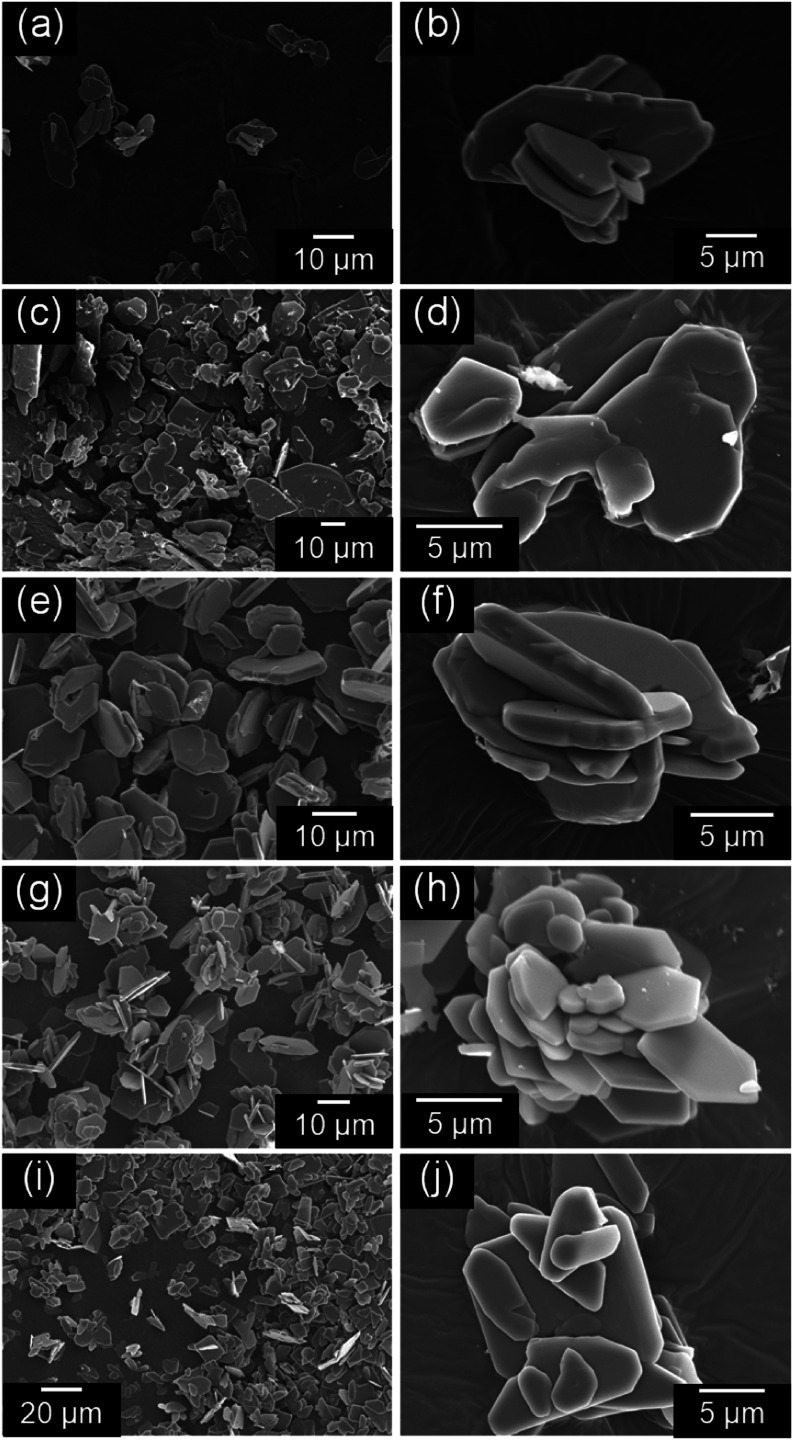
SEM images of NH_4_(Co_1−*x*_Mn_*x*_)PO_4_·H_2_O samples with *x* = 0 (a and b), 0.2 (c and d), 0.5 (e and f), 0.8 (g and h), and 1.0 (i and j).

Fig. 3 shows the SEM images of the NH_4_(Co_1−*x*_Mn_*x*_)PO_4_·H_2_O samples for *x* = 0–1.0. Platelike particles 10–20 μm in width were obtained for all the samples. The thickness of the platelike particles increases with *x* from 0 to 0.5, where the maximum thickness is *ca.* 1.5 μm (Fig. 3e and f). Further increase in *x* from 0.5 to 1.0 results in a decrease in thickness.

**Fig. 4:**
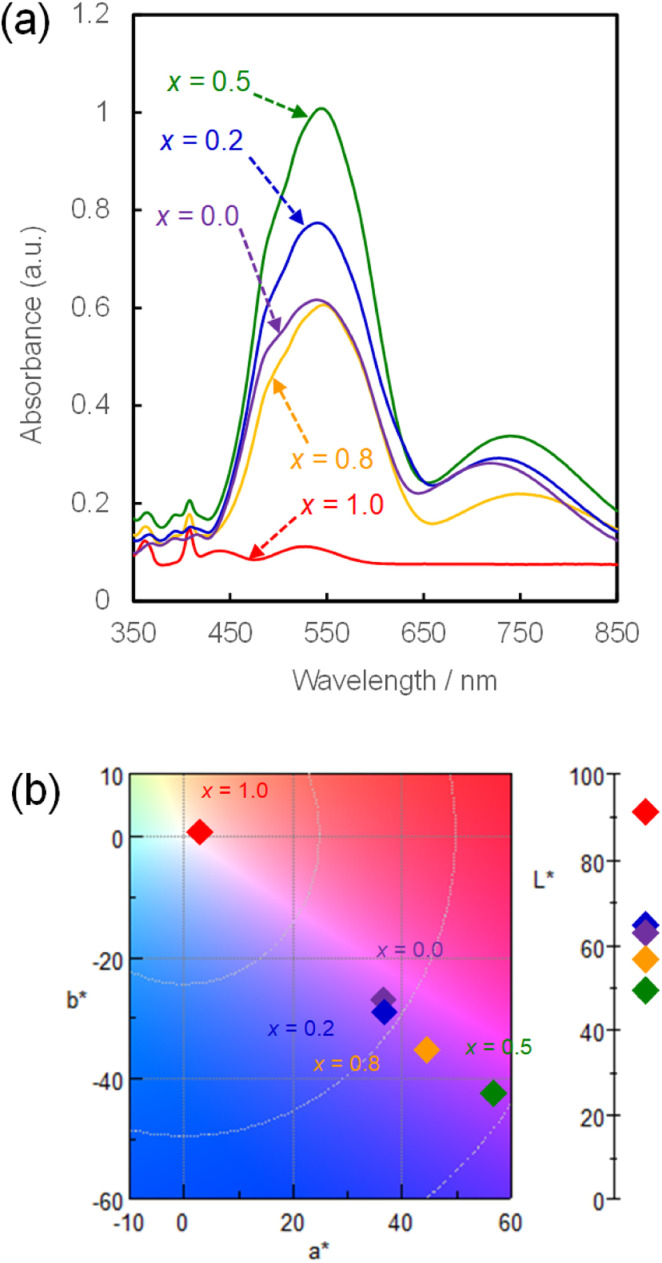
UV-Vis absorption spectra (a) and chromaticity diagram in L*a*b* colour space (b) for NH_4_(Co_1−*x*_Mn_*x*_)PO_4_·H_2_O samples with *x* = 0–1.0.

The UV-Vis absorption spectra of the NH_4_(Co_1−*x*_Mn_*x*_)PO_4_·H_2_O samples with *x* = 0–1.0 were measured, and the colour parameters in the L*a*b* colour space calculated from those spectra. Fig. 4a shows the UV-Vis absorption spectra of NH_4_(Co_1−*x*_Mn_*x*_)PO_4_·H_2_O samples with *x* = 0–1.0. Absorption peaks at approximately 550 and 750 nm are observed for the NH_4_CoPO_4_·H_2_O sample (*x* = 0), which are assigned to the ^4^T_1g_ → ^4^T_1g_(P) and ^4^T_1g_ → ^4^A_2g_(F) transition of the Co^2+^ ions in MO_6_ octahedra.^32^ Conversely, for the NH_4_MnPO_4_·H_2_O sample (*x* = 1.0), weak absorption peaks around 360, 400, 440 and 530 nm are observed, which may be caused by ^6^A_1g_(S) → ^4^T_1g_(G), ^6^A_1g_(S) → ^4^T_2g_(G), ^6^A_1g_(S) → ^4^A_1g_(G) + ^4^E_g_(G), and ^6^A_1g_(S) → ^4^T_2g_(D) transitions of Mn^2+^ ions with octahedral symmetry, respectively.^33^ All the photoabsorptions attributed to Co^2+^ and Mn^2+^ ions in the octahedral sites are also observed for NH_4_(Co_1−*x*_Mn_*x*_)PO_4_·H_2_O samples with *x* = 0.2–0.8. The *x* = 0.5 sample exhibited the highest absorbance, which may be attributed to the higher thickness of the platelike particles (Fig. 3e and f).

The L*a*b* colour parameters and the corresponding chromaticity diagram are shown in Table 2 and Fig. 4b, respectively. Increasing the Mn content from *x* = 0 to 0.5 increases a* and decreases b*. This means increases in the degrees of red and blue colouration, which results in a strong violet colour. A further increase in *x* from 0.5 to 1.0 weakens the violet colour, and the NH_4_MnPO_4_·H_2_O sample (*x* = 1.0) shows a very light pink colour. The colour characteristics, as determined by quantitative UV-Vis analysis, correspond well with the visual appearances of the samples (Fig. 1). Moreover, the NH_4_(Co_1−*x*_Mn_*x*_)PO_4_·H_2_O sample with *x* = 0.8 has a stronger violet colour than that of the NH_4_CoPO_4_·H_2_O (*x* = 0) sample.

**Table 2:** L*a*b* color parameters of the NH_4_(Co_1−*x*_Mn_*x*_)PO_4_·H_2_O samples with *x* = 0–1.0

*x*	Color parameters		
	L*	a*	b*
0.0	62.99	36.61	−27.28
0.2	64.39	37.01	−29.56
0.5	49.33	56.79	−42.65
0.8	56.53	44.63	−35.50
1.0	91.88	2.91	0.19

**Fig. 5:**
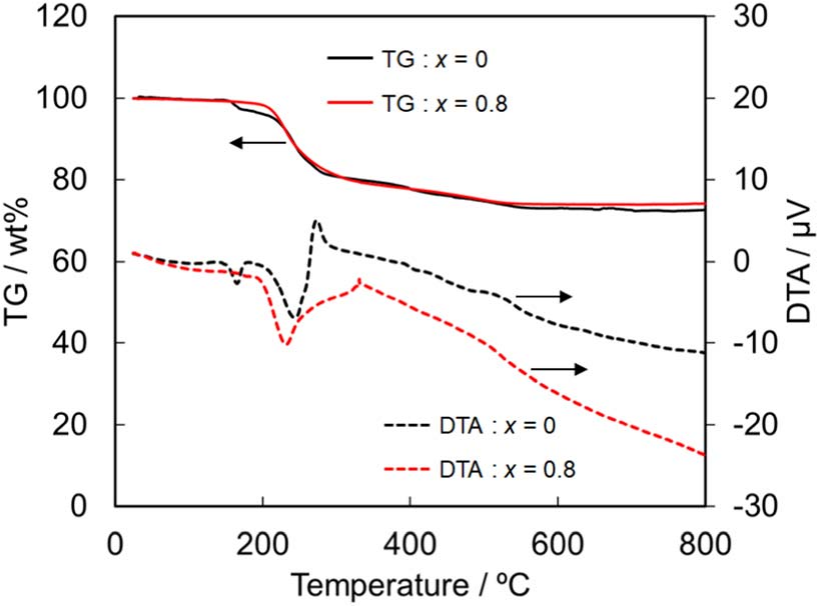
TG and DTA curves of NH_4_(Co_1−*x*_Mn_*x*_)PO_4_·H_2_O samples with *x* = 0 and 0.8.

Next, the heat and chemical resistances were compared between the NH_4_(Co_1−*x*_Mn_*x*_)PO_4_·H_2_O samples with *x* = 0 and 0.8. The heat resistance was evaluated by TG-DTA analysis. The TG-DTA curves of the samples with *x* = 0 and 0.8 are shown in Fig. 5. The weight loss and endothermic peaks are mainly observed at 200–300 °C for the both samples, and then the decrease in the weight gradually continued up to 500 °C. Moreover, the color of the both samples got light after the TG-DTA analysis. NH_4_CoPO_4_·H_2_O phase has been reported to be transformed to Co_2_P_2_O_7_ by the calcination.^34^ The total weight loss that detected in the present work was *ca.* 25% irrespective of samples, which well agreed with the theoretical value for the phase transition from NH_4_CoPO_4_·H_2_O to Co_2_P_2_O_7_. The TG-DTA curves suggest that the substitution of Co^2+^ by Mn^2+^ didn't affect the heat resistance.

The chemical resistance of the NH_4_(Co_1−*x*_Mn_*x*_)PO_4_·H_2_O samples was evaluated based on the colour change by the soaking in acidic and alkaline solutions. In the case of acidic conditions, the both samples completely dissolved in the HCl solutions. On the other hand, the soaking in NaOH solutions resulted in the partial dissolution and the colour change of the residual samples. ESI Fig. S3† shows the appearances of the residual samples with *x* = 0 and 0.8 after soaking in NaOH solutions. The violet colour of the sample with *x* = 0 was bleached and partially changed to blue, while the sample with *x* = 0.8 showed dark red-brown color after the NaOH soaking. As the results, for the both samples, the violet colour couldn't keep in the acidic and alkaline solutions.

As mentioned above, the violet colour of the NH_4_CoPO_4_·H_2_O particles with *x* = 0 was maintained upon substitution of Co^2+^ with Mn^2+^ ions up to *x* = 0.8. On the other hand, the heat and chemical resistances of NH_4_CoPO_4_·H_2_O phase were not improved by the substitution by Mn^2+^ ions. However, the addition of Mn^2+^ ions didn't degrade the resistances. These results present an 80% cobalt resource saving with no decline in colour.

## Conclusions

We hydrothermally prepared NH_4_(Co_1−*x*_Mn_*x*_)PO_4_·H_2_O particles and investigated the effects of replacing Co^2+^ ions with Mn^2+^ ions on the colour of the resulting materials. Single-phase NH_4_(Co_1−*x*_Mn_*x*_)PO_4_·H_2_O solid-solution particles were obtained by hydrothermal treatment at 150 °C, and the [Mn^2+^]/([Co^2+^] + [Mn^2+^]) mole ratios of the samples were controlled by changing the MnCl_2_ and CoCl_2_ concentrations in the precursor solutions. The violet colour of ammonium cobalt phosphate (NH_4_CoPO_4_·H_2_O) particles was maintained when their Mn content was increased to *x* = 0.8, thus achieving an 80% saving of cobalt in the preparation of violet pigments. These simple synthetic route for dittmarite-group solid-solution materials would be beneficial for many researchers in material fields.

## Author contributions

Hiroaki Uchiyama conceived of the study, designed the study, and drafted the manuscript; Saori Tachibana carried out the sample synthesis and characterization. All authors have given approval to the final version of the manuscript.

## Conflicts of interest

There are no conflicts to declare.

## Supplementary Material

RA-014-D3RA07387D-s001
