# Supplementary material for: Hydrothermal synthesis of dittmarite-group NH4(Co1−xMnx)PO4·H2O particles as inorganic violet pigments
Source: RSC Adv. 2024 Jan 8;14(3):1939–43. doi: 10.1039/d3ra07387d (PMC10772953; doi:10.1039/d3ra07387d)
Supplement: RA-014-D3RA07387D-s001 [file RA-014-D3RA07387D-s001.pdf]

Electronic Supplementary Material (ESI) for RSC Advances.
This journal is © The Royal Society of Chemistry 2023

Supplementary information

Hydrothermal synthesis of dittmarite-group $NH_4(Co_{1-x}Mn_x)PO_4 \cdot H_2O$ particles as inorganic□violet pigments

Hiroaki Uchiyama* and Saori Tachibana

Corresponding Author

Hiroaki Uchiyama, Department of Chemistry and Materials Engineering, Kansai University, 3-3-35 Yamate-cho, Suita, 564-8680, Japan. E-mail: h_uchi@kansai-u.ac.jp (H. Uchiyama)

Authors

Saori Tachibana, Department of Chemistry and Materials Engineering, Kansai University, 3-3-35 Yamate-cho, Suita, 564-8680, Japan

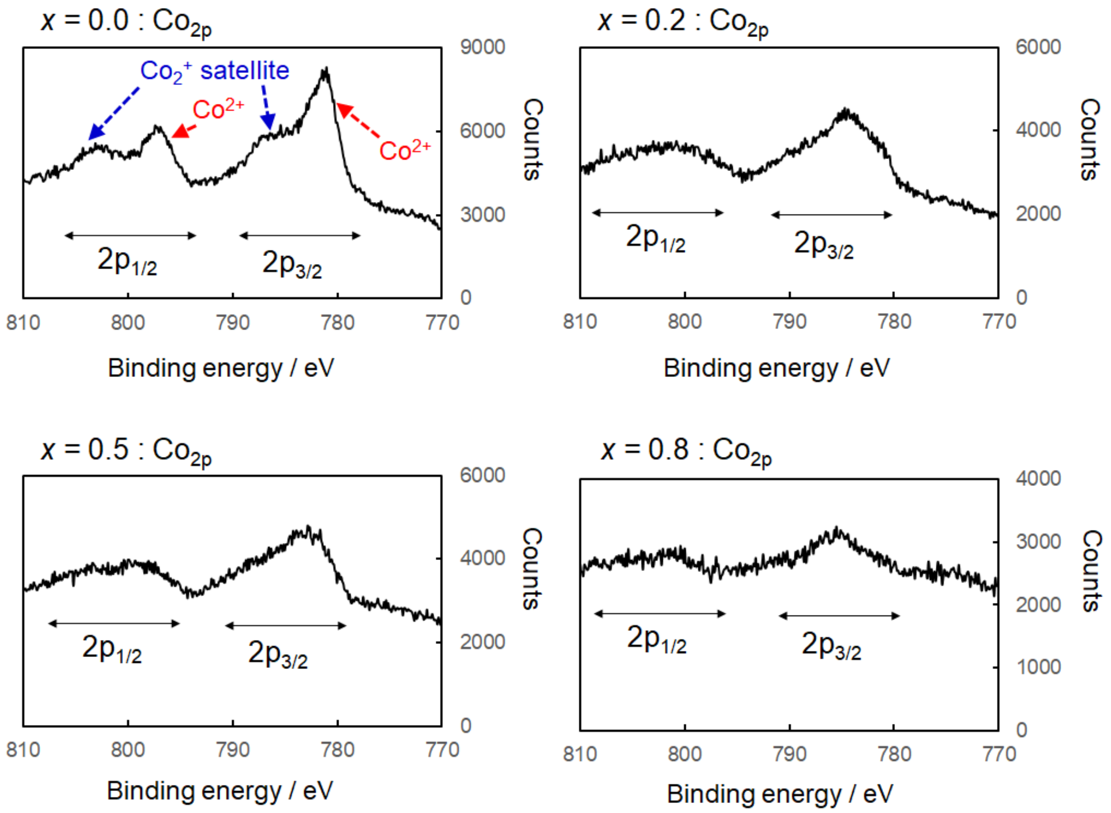

Fig. S1 XPS spectra (Co_{2p}) of the $NH_4(Co_{1\text{-}x}Mn_x)PO_4{\cdot}H_2O$ samples with x = 0–0.8.

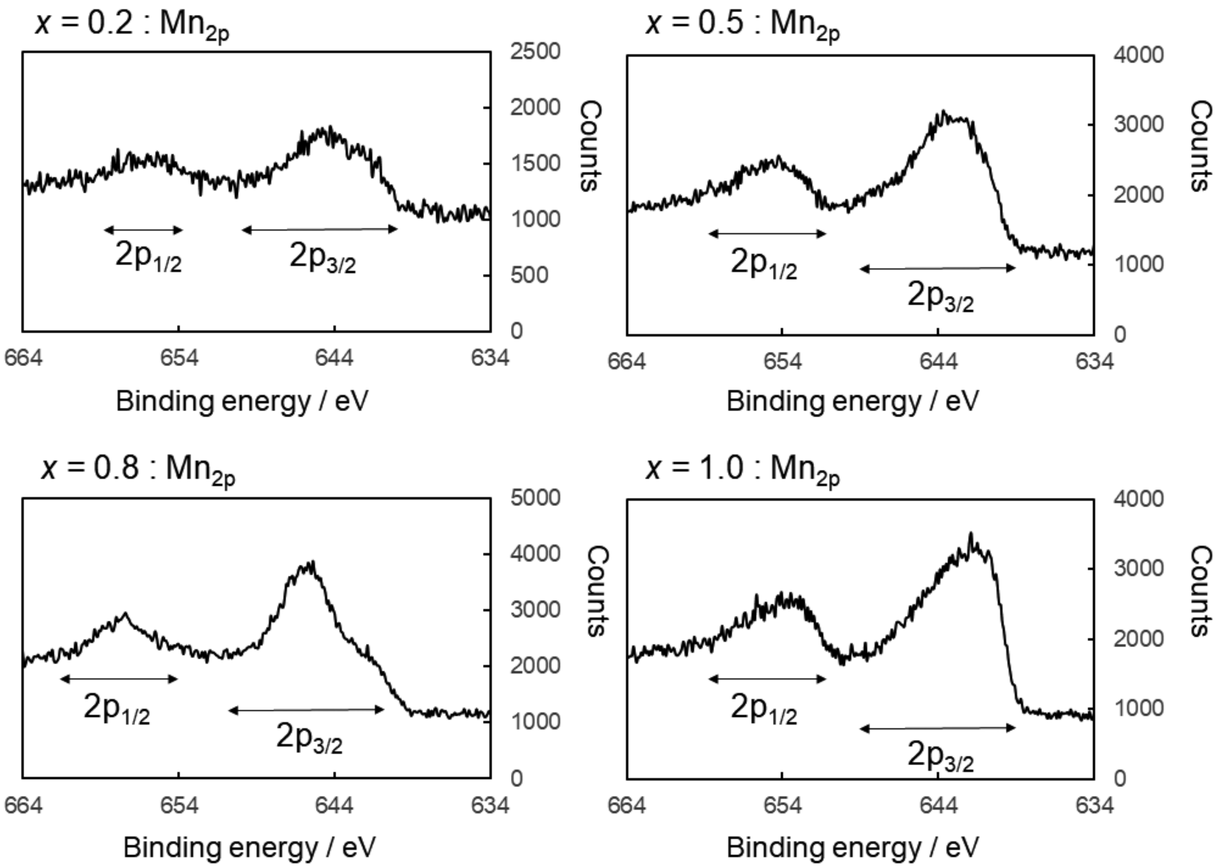

Fig. S2 XPS spectra (Mn_{2p}) of the $NH_4(Co_{1-x}Mn_x)PO_4 \cdot H_2O$ samples with x = 0.2–1.0.

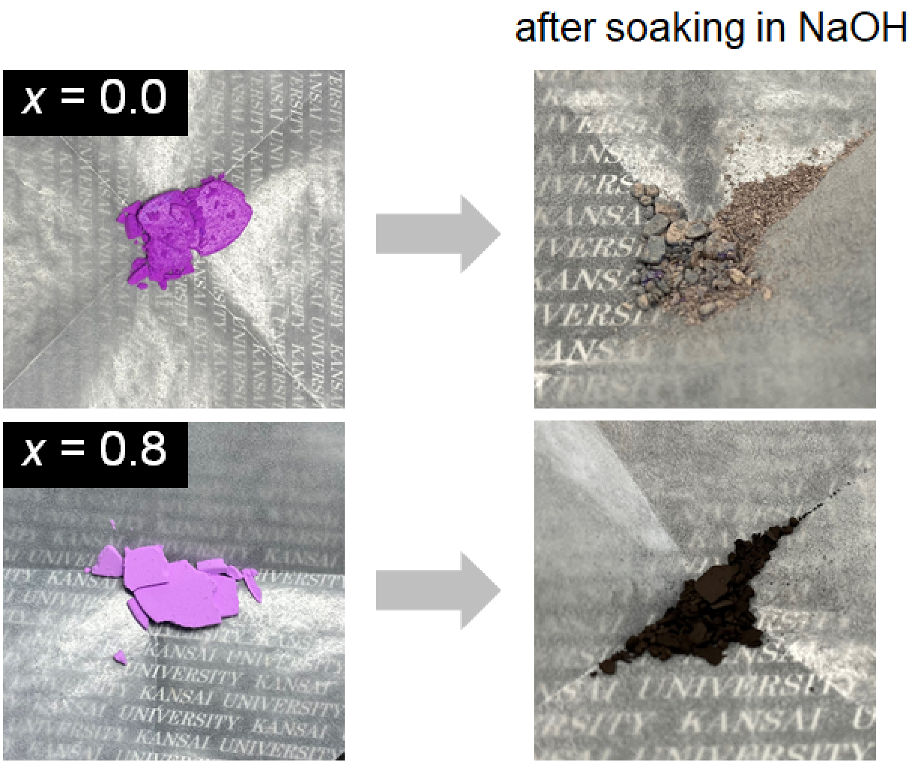

Fig. S3 Appearances of $NH_4(Co_{1-x}Mn_x)PO_4 \cdot H_2O$ samples with x = 0 and 0.8 before and after soaking in NaOH solutions.
